# Being Overweight or Obese Is Associated with an Increased Platelet Reactivity Despite Dual Antiplatelet Therapy with Aspirin and Clopidogrel

**DOI:** 10.1007/s10557-022-07325-z

**Published:** 2022-02-25

**Authors:** Marianna Puccini, Christian Rauch, Kai Jakobs, Julian Friebel, Adel Hassanein, Ulf Landmesser, Ursula Rauch

**Affiliations:** 1 Charité Center 11–Department of Cardiology, Charité–University Medicine, Berlin, Germany; 2 DZHK (German Centre for Cardiovascular Research), Partner Site Berlin, Berlin, Germany; 3 Berlin Institute of Health, Berlin, Germany; 4 Department of Cardiology, Charité–Universitätsmedizin Berlin, Campus Benjamin Franklin, Hindenburgdamm 30, 12200 Berlin, Germany

**Keywords:** Overweight, Obesity, Platelet function, Dual antiplatelet therapy, Aggregometry

## Abstract

**Purpose:**

Obese patients exhibit an overall increased platelet reactivity and a reduced sensitivity to antiplatelet therapy. The aim of this study is to evaluate the platelet reactivity measured by impedance aggregometry in overweight and obese patients and chronic coronary syndrome (CCS) that were treated with dual antiplatelet therapy (DAPT).

**Methods:**

Platelet aggregation was assessed by impedance aggregometry in patients with CCS receiving DAPT (aspirin plus clopidogrel). We compared the platelet reactivity in patients with a normal weight versus overweight or obese patients. Furthermore, the correlation between the body mass index (BMI) and adenosine diphosphate- (ADP-) or thrombin receptor-activating peptide- (TRAP-) dependent platelet aggregation was analyzed.

**Results:**

64 patients were included in the study of which 35.9% were patients with normal weight. A higher ADP- and TRAP-dependent platelet reactivity was observed in overweight and obese patients (ADP: median 27 units (U) [IQR 13–39.5] vs. 7 U [6–15], *p* < 0.001 and TRAP: 97 U [73–118.5] vs. 85 U [36–103], *p* = 0.035). Significant positive correlations were observed between agonist-induced platelet reactivity and BMI.

**Conclusion:**

Despite the use of DAPT, a higher platelet reactivity was found in overweight and obese patients with CCS. If these patients will benefit from treatment with more potent platelet inhibitors, it needs to be evaluated in future clinical trials.

## Introduction

Obesity is an important modifiable risk factor that contributes to the pathogenesis of diabetes, cancer, and cardiovascular diseases (CVD), among other entities [1, 2]. It is also a risk factor for arterial and venous thrombotic events. For patients with a high ischemic risk and a low bleeding risk, the actual guidelines recommend after a percutaneous coronary intervention that the dual antiplatelet therapy (DAPT) be prolonged for more than 12 months [2].

The link between obesity and CVD can be explained by the presence of a pro-inflammatory and pro-thrombotic state in this group of patients [1, 3]. Possible mechanisms that may explain the increased thrombotic risk in obese patients are due to altered expression of profiles of proteins of the coagulation and fibrinolytic cascade as well as an increased platelet reactivity [4]. The change in platelet function in obesity is reflected by elevated levels of platelet activation markers such as mean platelet volume, thromboxane B2 metabolites, circulating levels of platelet microparticles, soluble p-selectin, and platelet derived CD40L [5].

Furthermore, a reduced sensitivity to antiplatelet therapy has been observed in obesity in patients receiving monotherapy with either aspirin or clopidogrel [1, 4, 6]. In several studies, it has already been reported that obese and overweight patients exhibit a higher platelet aggregability [7, 8]. However, little is available regarding the platelet reactivity measured with impedance aggregometry in obese and overweight patients while receiving DAPT. This method is a whole blood bedside test, which allows to evaluate the platelet function in an easy and quick way.

The present study aims to evaluate the difference of the adenosine diphosphate- (ADP-) and thrombin receptor-activating peptide- (TRAP-) induced platelet aggregation measured by impedance aggregometry between overweight and obese patients with chronic coronary syndrome (CCS) in comparison with those CCS patients who have a normal body mass index (BMI). All patients received DAPT with aspirin and clopidogrel. Furthermore, the association between platelet reactivity and BMI will be also assessed.

## Material and Methods

### Study Design and Patient Population

In this cross-sectional study, we analyzed data of consecutive patients who were admitted to the Department of Cardiology, Campus Benjamin Franklin, Charité Universitätsmedizin Berlin. All patients had a known CCS and received aspirin 100 mg/day and clopidogrel 75 mg/day. Impedance aggregometry was done in all patients with a multi-electrode aggregometry (MEA, Multiplate® Analyzer, Roche Diagnostics). Patients with congenital coagulopathy and on treatment with oral anticoagulants, prasugrel, or ticagrelor were excluded from the study. We compared the groups of patients with a normal weight (BMI 18.5–25 kg/m^2^) versus patients with a BMI ≥ 25 kg/m^2^. The study was conducted in accordance with the ethical standards of the Helsinki Declaration of 1975.

### Data Collection

The following data was collected: demographics (age, gender, and BMI), cardiovascular risk factors (hypertension, hypercholesterolemia, and active smoking), co-morbidities (chronic obstructive pulmonary disease, diabetes, and previous myocardial infarction), as well as the use of aspirin and clopidogrel. The serum creatinine level (mg/dL), leucocytes (/nl), hemoglobin (g/dL), platelet count (/nL), and mean platelet volume (fl) were also collected.

### Platelet Function Tests

ADP- and TRAP-induced platelet aggregation were assessed by impedance aggregometry using a MEA called Multiplate® Analyzer (Roche Diagnostics, Mannheim, Germany) [9]. For the measurement, patients’ blood was drawn and collected in hirudin-coated tubes. 300 μL of whole blood was then added to preheated 0.9% NaCl. Platelet aggregation was induced by platelet activation with adenosine diphosphate (ADP, final concentration 6.4 µM) or thrombin receptor-activating peptide (TRAP, final concentration 32 µM). The activated platelets adhere to the electrode´s surface and aggregate and thereby increase the resistance between the two electrodes. The degree of platelet aggregation is recorded for 6 min and is expressed as units of area under the curve plotted against time [10].

### Statistics

Continuous variables were analyzed by Mann–Whitney *U* test and presented as median with interquartile ranges. Categorical variables were analyzed with a Fisher’s exact test and are presented as frequencies and percentages. To analyze the correlation between continuous variables, the Spearman’s correlation was performed. All tests were two-sided; 95% confidence intervals were used, and a *p*-value < 0.05 was considered statistically significant. The statistical analyses were performed using the software IBM SPSS Statistics version 27.0, and the graphs were generated with GraphPad Prism 8.4.3.

## Results

A total of 64 patients were included into the analysis, of which 23 (35.9%) had normal weight and 41 (64.1%) were overweight or obese (BMI ≥ 25 kg/m^2^). No significant differences in baseline characteristics were observed between the two groups of patients (Table 1).

**Table 1:** Demographics and clinical characteristics of the patient population

	Overall (*n* = 64)	BMI 18.5–25 kg/m^2^ (*n* = 23)	BMI > 25 kg/m^2^ (*n* = 41)	*p*
Age^*^	65.5 (59–72.8)	62 (55–72)	67 (61.5–73)	0.320
Gender (male)	47 (73.4%)	15 (65.2%)	32 (78%)	0.377
BMI (kg/m^2^)^*^	26.3 (24.1–29.1)	23.7 (22–24.2)	28.0 (26.95–30.45)	< 0.001
Hypertension	63 (98.4%)	22 (95.7%)	41 (100%)	0.359
Hypercholesterolemia	62 (96.9%)	23 (100%)	39 (95.1%)	0.532
Active smoking	23 (35.9%)	8 (34.8%)	15 (36.6%)	1.0
Previous ischemic stroke	5 (7.8%)	2 (8.7%)	3 (7.3%)	1.0
Previous MI	39 (60.9%)	13 (56.5%)	26 (63.4%)	0.605
Type 2 diabetes mellitus	16 (25%)	3 (13.0%)	13 (31.7%)	0.136
COPD	7 (10.9%)	2 (8.7%)	5 (12.2%)	1.0
Aspirin	64 (100%)	23 (100%)	41 (100%)	–
Clopidogrel	64 (100%)	23 (100%)	41 (100%)	–
ACE inhibitor/ARB	55 (85.9%)	17 (73.9%)	38 (92.7%)	0.060
Betablocker	53 (82.8%)	19 (82.6%)	34 (82.9%)	1.0
Calcium chanel blockers	13 (20.3%)	3 (13.0%)	10 (24.4%)	0.346
Diuretics	36 (56.3%)	9 (39.1%)	27 (65.9%)	0.065
Aldosteron antagonists	7 (10.9%)	2 (8.7%)	5 (12.2%)	1.0
Statins	59 (92.2%)	19 (82.6%)	40 (97.6%)	0.052
Serum creatinin (mg/dL)^*^	0.94 (0.84–1.12)	0.92 (0,83–1.14)	0.94 (0.87–1.11)	0.817
Leucocytes (/nL)^*^	7.07 (5.61–8.85)	6.68 (5.4–8.2)	7.38 (5.79–9.07)	0.454
Hemoglobin (g/dL)^*^	14.05 (12.8–15.13)	13.8 (12–14.8)	14.2 (12.9–15.3)	0.278
Platelet count (n/L)^*^	221 (181.5–262.5)	219 (171–246)	221 (183–267.5)	0.520
MPV (fl)^*^	10 (10–11)	10 (10–11)	10 (10–11)	0.507

^*^Continuous data presented as median (IQR). *BMI* body mass index, *MI* myocardial infarction, *COPD* chronic obstructive pulmonary disease, *ACE* angiotensin converting enzyme, *ARB* angiotensin II receptor blocker, *MPV* mean platelet volume

**Fig. 1:**
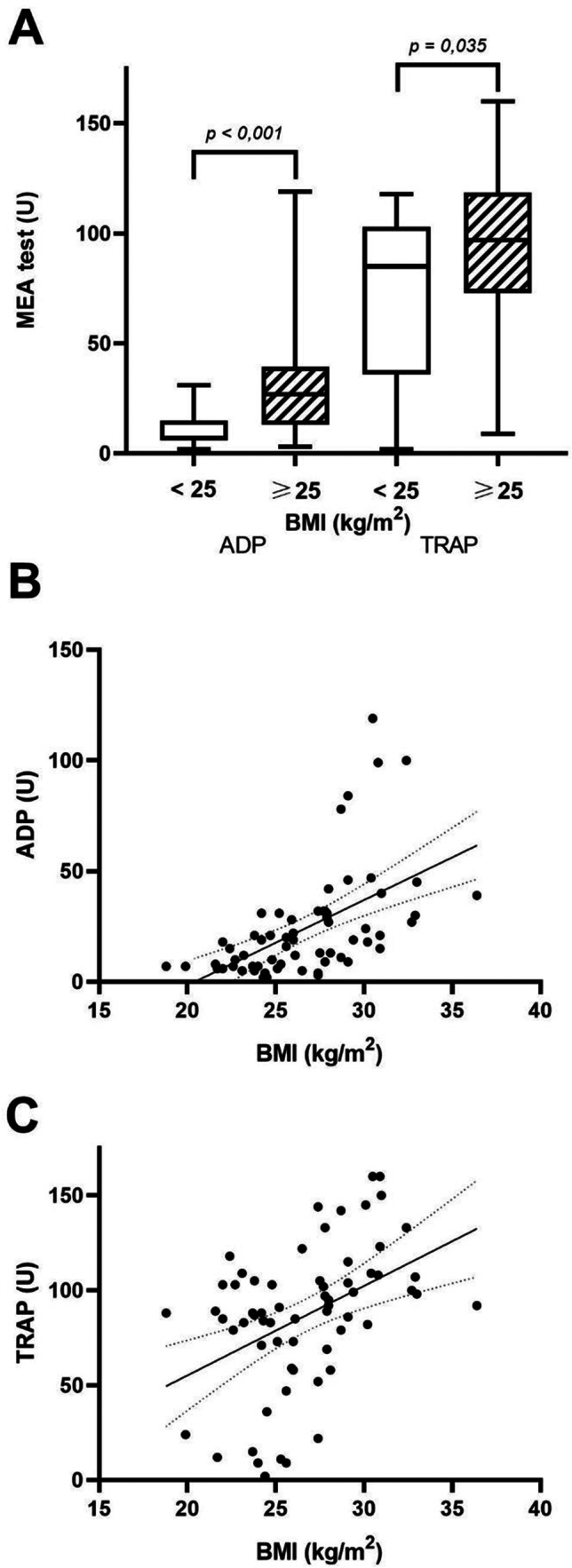
Platelet aggregation measured by impedance aggregometry in patients with BMI < 25 kg/m^2^ (*n* = 23) and BMI ≥ 25 kg/m^2^ (*n* = 41) and Spearman’s correlation between platelet reactivity and BMI. **A** ADP indicates the ADP-induced platelet reactivity and TRAP-induced platelet reactivity. **B** Correlation between ADP-induced platelet reactivity as a function of the body mass index (*r* = 0.643, *p* < 0.001). **C** Correlation between thrombin-induced platelet reactivity as a function of the body mass index (*r* = 0.453, *p* < 0.001)

The ADP-induced platelet aggregation was higher in patients with a BMI ≥ 25 kg/m^2^ compared to those with a BMI between 18 and 25 kg/m^2^ (median ADP 27 U [13–39.5] vs. 7 U [6–15], *p* < 0.001). In addition, the TRAP-induced platelet aggregation was also increased in patients in the higher BMI group compared to those in the lower BMI group (median TRAP 97 U [73–118.5] vs. 85 U [36–103], *p* = 0.035) (Fig. 1A). These findings point to the presence of heightened platelet reactivity in CCS patients that are overweight or obese, despite concurrent treatment with aspirin and clopidogrel.

When analyzing the association between the ADP-induced platelet aggregation and BMI, a statistically significant positive correlation between these variables was found (*r* = 0.643, *p* < 0.001, Fig. 1B). A positive correlation was also present between TRAP-induced platelet reactivity and BMI, indicating a relationship between platelet function and body mass (*r* = 0.453, *p* < 0.001, Fig. 1C).

## Discussion

Our study shows that patients with chronic coronary syndrome (CCS) and a BMI ≥ 25 kg/m^2^ have a higher ADP- and TRAP-induced platelet reactivity while on DAPT (with aspirin and clopidogrel) than those with a BMI < 25 kg/m^2^. When analyzing the association between ADP- or TRAP-induced platelet reactivity and BMI, a positive correlation was seen with both.

The persistent “high on treatment platelet reactivity” (HTPR) has been associated with recurrence of the thrombotic events, stent thrombosis, and major cardiovascular events [11]. Therefore, identifying the variable individual risk factors for HTPR might help to personalize the treatment of these patients [12].

Since obesity has become a major public health problem, special attention has been given to assess whether the BMI has an influence on the platelet reactivity. This aspect is important to take into account when assessing the antiplatelet therapy for patients because we could potentially influence its low sensitivity by lowering the BMI.

Several studies have shown a higher platelet reactivity in obese patients in comparison to normal weight patients [6, 7]. However, in the majority of these previous studies, the patients were either on single antiplatelet therapy or they were not treated with a platelet inhibitor at all. We previously demonstrated an increased platelet reactivity in postmenopausal women and in patients with type 2 diabetes despite the treatment with DAPT [10, 13]. Nonetheless, the BMI was not evaluated as an important confounder.

In our study, we evaluated the difference of the ADP- and TRAP-dependent platelet reactivity measured with MEA in overweight and obese patients in comparison to patients with normal weight while receiving treatment with DAPT with aspirin and clopidogrel. Our results are consistent with the known effect of obesity and being overweight on platelet reactivity suggesting that even when being on DAPT, it should be considered to assess the response to platelet inhibition in these patients. A study that evaluated platelet reactivity in obese patients, but used different platelet function tests, showed that subjects with a higher BMI on DAPT with either aspirin and clopidogrel or prasugrel had a higher prevalence of HTPR [14]. However, another study done by Deharo et al. could not document any difference in platelet reactivity between obese and overweight patients in comparison to those with normal weight while on DAPT with aspirin and ticagrelor [15], suggesting that it is likely that this group of patients had a benefit from a stronger platelet inhibition.

Regarding the association between BMI and platelet function, some other studies have also reported a weak but significant correlation while on DAPT as well as while on monotherapy [7, 16].

Looking at our patient population in the overweight and obesity group, it is important to note that these patients were mainly overweight. These findings reflect that an increased platelet reactivity plays an important role not only for obese patients but also for overweight patients, consistent to what previous studies have shown, even though the previous studies usually use the obesity cut-off [7]. This stresses the importance of evaluating the antiplatelet therapy not only in obese but also on overweight patients, since a HTPR predicts poor clinical outcomes after an intervention.

## Conclusions

Despite the use of DAPT, overweight and obese patients with CCS have a higher platelet reactivity than patients with a normal BMI. Further prospective studies are needed to assess whether treatment with more potent platelet inhibitor may improve the clinical outcome in these patients.

## Data Availability

Please contact the authors for further data request.
